# Acetylcholinesterase Biosensor Based on Functionalized Renewable Carbon Platform for Detection of Carbaryl in Food

**DOI:** 10.3390/bios12070486

**Published:** 2022-07-03

**Authors:** Erik W. Nunes, Martin K. L. Silva, Jesús Rascón, Damaris Leiva-Tafur, Rainer M. L. Lapa, Ivana Cesarino

**Affiliations:** 1 School of Agriculture, São Paulo State University (UNESP), Botucatu 18610-034, Brazil; erik.weber@unesp.br (E.W.N.); martin.leme@unesp.br (M.K.L.S.); 2 Instituto de Investigación para el Desarrollo Sustentable de Ceja de Selva (INDES-CES), Universidad Nacional Toribio Rodríguez de Mendoza de Amazonas (UNTRM), Chachapoyas 01001, Peru; jesus.rascon@untrm.edu.pe (J.R.); damaris.leiva@untrm.edu.pe (D.L.-T.); 3 Instituto de Investigación en Ganadería y Biotecnología (IGBI), Universidad Nacional Toribio Rodríguez de Mendoza de Amazonas (UNTRM), Chachapoyas 01001, Peru; rainer.lopez@untrm.edu.pe; 4 Facultad de Ciencias de la Salud (FACISA), Universidad Nacional Toribio Rodríguez de Mendoza de Amazonas (UNTRM), Chachapoyas 01001, Peru

**Keywords:** electrochemical biosensor, renewable carbon, carbaryl, acetylcholinesterase enzyme, food safety

## Abstract

Enzymatic electrochemical biosensors play an important role in the agri-food sector due to the need to develop sustainable, low-cost, and easy-to-use analytical devices. Such biosensors can be used to monitor pathogens, endocrine disruptors, and pesticides, such as carbaryl, widely used in many crops. The use of renewable carbon (RC) sources, provided from biomass pyrolysis has been often applied in the fabrication of such sensors. This material is a great candidate for biosensor fabrication due to the presence of surface functional groups, porosity, and moderate surface area. This work describes the functionalization of RC material through an acid treatment with a sulfonitric solution HNO_3_/H_2_SO_4_ (1:3) and the resulting material was characterized by scanning electron microscopy. The obtained RC functionalized (RCF) and the acetylcholinesterase enzyme (AChE) were applied in the construction of the electrochemical biosensor on glassy carbon (GC) electrode and used to detect carbaryl in apple samples. The GC/RCF/AChE biosensor was able to detect the carbaryl pesticide from 5.0 to 30.0 nmol L^−1^, displaying a LOD of 4.5 nmol L^−1^. The detection of carbaryl in apple samples presented recoveries between 102.5 to 118.6% through the standard addition method. The proposed biosensor is a promising renewable tool for food safety.

## 1. Introduction

The exponential population growth, mainly during the last century, has increased the demand for the intrinsic factors that provide life support. Among the most important sectors, the agro-industrial and forestry supply both food and raw materials to the most varied areas. To sustain this growing demand, synthetic products (such as chemical pesticides) are widely used to control pests and optimize production. However, the inappropriate use of these compounds and their side effects on non-target organisms/species, along with the poor waste management of these sectors (framed on the scale of millions of tons per year) raise important problems, extensively discussed in the literature [1,2].

Currently, two classes of pesticides (organophosphates and carbamates) have gained prominence. Both have a broad biological activity by inhibiting the enzyme acetylcholinesterase (AChE), responsible for the catalysis of acetylcholine (ACh). The neurotransmitter ACh acts in autonomous ganglia, neuromuscular junctions, and synapses in the central nervous system [3,4]. The accumulative contamination in the human body is extensively described in the literature as anti-AChE intoxication, causing restlessness, partial or generalized seizures, cyanosis, and coma, among other problems related to the dysfunction of neurological and motor systems [5]. In Brazil, carbaryl has been widely used as a pesticide in apple farms, with its limit concentration of carbaryl stipulated at 2.0 mg/kg in apple crops by the National Health Surveillance Agency (ANVISA) [6].

Among the most frequent approaches for detecting pesticide residues, chromatographic techniques, such as high-efficiency gas and liquid chromatography, or even spectroscopic ones, have attracted significant attention in research [7]. However, the analyzed matrices must be treated due to the high interferent content. This results in high operational costs, additional equipment, the need for specialized operators, and restrictions on in situ analysis.

In order to circumvent these limitations, electroanalytical biosensors have been standing out for offering a fast response, simplicity/convenience, low cost, good selectivity, high specificity, and in situ analysis for the detection and quantification of carbamates and organophosphate pesticides, mainly in the environmental, food and clinical [7,8].

An enzymatic biosensor combines an enzyme with a transducer to give a signal proportional to the target analyte concentration. By monitoring the cathodic peak height of reactions, voltammetric sensors, such as those based on differential pulse voltammetry, square wave voltammetry, and linear sweep voltammetry, can be used to quantify the concentration effects of specific analytes. Nevertheless, inhibitory biosensor systems might face challenges such as non-uniform dispersion of immobilized enzyme in the host matrix, and the low stability and bioreactivity of the enzyme [9,10,11].

Electrochemical transducers are strongly associated with the development of biosensors, highlighting sensors based on the mechanism of cholinesterase enzyme inhibition. Enzymatic biosensors can explore the inhibition reaction of AChE, as well as its substrate, acetylcholine (AChI), monitoring the pesticides of the carbamate class [12]. In a process occurred by phosphorylation or carbamylation, the carbamates bind to the active stearic site of the enzyme, inhibiting the biocatalytic activity by blocking the serine residue in the catalytic triad [12]. Previous research optimized the main parameters and conditions, avoiding problems such as interference from other reactive agents and poor detection limits [8].

One of the most important aspects of the sensor’s development is the selection of the surface-modification material [13,14]. Recently, carbon-based nanomaterials (e.g., graphene and carbon nanotubes) have been extensively explored as a modifier in electrodes owing to their good synergy with other materials [15]. However, due to the complexity of their synthesis and characterization, simpler and lower-cost carbon-based alternatives such as renewable carbon (RC) are investigated [16,17]. In parallel, its classification as a renewable product adds value to this class of residues [18,19].

As low-value agricultural wastes (such as hull or chaff) are sent to landfills when no agricultural application exists, the production of the composite material RC (also referred as “biocarbon” or “biochar”) is a promising use of this potential waste product. RC is a carbon-rich by-product of bio-oil production, derived from biomass pyrolysis [20]. The structural and functional properties of RC are influenced by the biomass precursor or the conditions of the pyrolysis as temperature, rate of heating, and the variable ratio of combustible gases, among others [21,22]. RC is mostly used as an efficient sorbent for the treatment of contaminants, as well as a co-adjuvant in the exchange of cations and the absorption of nutrients in planting regions, based on its contact surface and highly functionalized composition [23]. 

Similar to other carbon-based materials, there is a growing interest in studying its electroanalytical characteristics, as RC production and implementation only require a simple infrastructure and methodology [17]. In order to enhance the kinetics of the electrochemical processes, improving the sensor development and its characteristics, many articles describe a modification step for the material used [24,25]. In the case of RC, chemical agents are usually used for its surface modification, such as sodium hydroxide, and nitric acid, among others [25,26]. 

In a previous, study of our group [25], the RCF material was characterized and applied in the detection of inorganic and organic chemical species. Hence, the RCF material could be applied in biosensing development, and since there are still few works in the literature reporting electrochemical biosensors for pesticide detection in complex matrices such as food, the investigation of such matter is required. 

For the first, the electrochemical biosensor GC/RCF/AChE was characterized and applied in the detection of carbaryl in apple samples. This provides a reference/guideline for utilizing a renewable carbon-based material, derived from residues, with low complexity for their synthesis and characterization. Moreover, the results expand the applications of renewable carbon towards the development of novel electrochemical sensors.

## 2. Materials and Methods

### 2.1. Reagents and Apparatus

Acetylcholinesterase enzyme (EC:232-559-3) from Electrophorus electricus (electric eel) Type VI-S (lyophilized powder, 200–1000 units/mg protein), Acetylthiocholine iodide (AChI), Carbaryl PESTANAL^®^ were obtained from Sigma-Aldrich (Darmstadt, Germany). Electrochemical experiments were performed in a conventional three-electrode system in the Autolab 128N electrochemical system. A bare glassy carbon (GC) electrode was modified with the renewable carbon and AChE enzyme (GC/RC/AChE), or functionalized renewable carbon and AChE enzyme (GC/RCF/AChE) as the working electrode, the Ag/AgCl/ KCl (3.0 mol L^−1^) as the reference electrode, and a platinum plate as the auxiliary electrode. The resulting data were analyzed via NOVA 2.1 software.

### 2.2. Renewable Carbon Functionalization and Biosensor Fabrication

**Figure 1:**
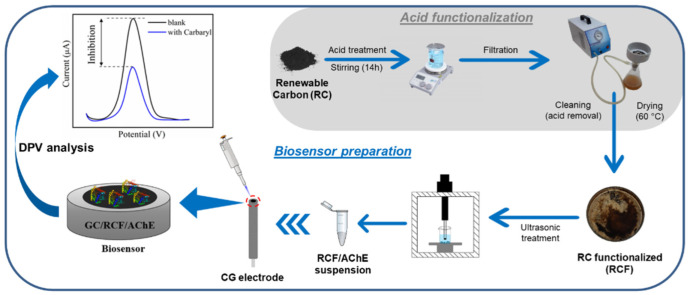
Schematic representation of RC functionalization and biosensor construction.

The RC sample obtention was fully described in our previous work [25]. RC was functionalized through a chemical oxidation step with a sulfonitric solution HNO_3_/H_2_SO_4_ (1:3), under vigorous stirring. The obtained RCF was filtered through a nylon membrane (0.45 µm) and washed several times for acid removal. RCF was then dispersed with an ultrasonic probe at 1.0 mg mL^−1^ in ultrapure water. Prior to modification, the RC and RCF suspensions were sonicated for 2 sets of 10 min. For the biosensor fabrication, 1 mL of RC or RCF was mixed with 200 µL (containing 80 U) of AChE enzyme suspension in PBS solution (pH 7.0). An aliquot of 10 µL of the mix was drop cast onto the surface of polished and cleaned GC electrodes, and let dry at room temperature. Figure 1 summarizes the steps of the acid RCF and biosensor fabrication. 

### 2.3. Preparation of Real/Apple/Fruit Samples for Carbaryl Analysis

For the determination of carbaryl in real samples, apple samples were acquired from a local supermarket (in the city of Botucatu-SP, Brazil) and selected as real samples. Thus, 100 g of apple (hull and seed included) were grounded, using a blender in 100 mL of a 0.2 mol L^−1^ PBS solution (pH 7.5), centrifuged for 5 min at 3500 rpm and its supernatant was collected and used for the electrochemical experiments. Differential Pulse Voltammetry (DPV) experiments were performed with the apple samples fortified with 10 nmol L^−1^ of carbaryl. Three successive additions of the standard were performed to quantify the analyte by the standard addition method. The pesticide concentration was detected by the calibration curve method and the recovery percentage was calculated.

### 2.4. Electrochemical Experiments

The DPV experiments evaluated the electrochemical responses of GC/RCF/AChE biosensor from −0.3 to 0.5 V, at a scan rate of 50 mV s^−1^ in 0.2 mol L^−1^ PBS solution (pH 7.0) containing 50.0 µmol L^−1^ of AChI. The incubation time for AChI and carbaryl pesticide standard solutions was 5 min. The analytical curve was constructed upon the inhibition effect of the anodic peak current (*I_pa_*) of AChI in the presence of carbaryl. The inhibition (I (%)) of the AChE enzyme was determined by measuring the anodic peak current of dithio-bis-choline before (*I_i_*) and after (*I_i_*) incubation with the catalytic inhibitor [12] carbaryl using the following Equation (1):

(1) $$I\left(\%\right)=\frac{I_{0}-I_{i}}{I_{0}}\times 100$$

The limit of detection (LOD) and limit of quantification (LOQ) were calculated using relation with the standard deviation of blank measurements (α) and the slope of the corresponding calibration curve [7], as represented in the Equations (2) and (3):

(2) $$\mathrm{LOD}=\frac{3.3\;\times \;\alpha }{\text{slope}}\;\times \;100$$

(3) $$\mathrm{LOQ}=\frac{10\;\times \;\alpha }{\text{slope}}\;\times \;100$$

## 3. Results and Discussion

### 3.1. Morphological Characterization

**Figure 2:**
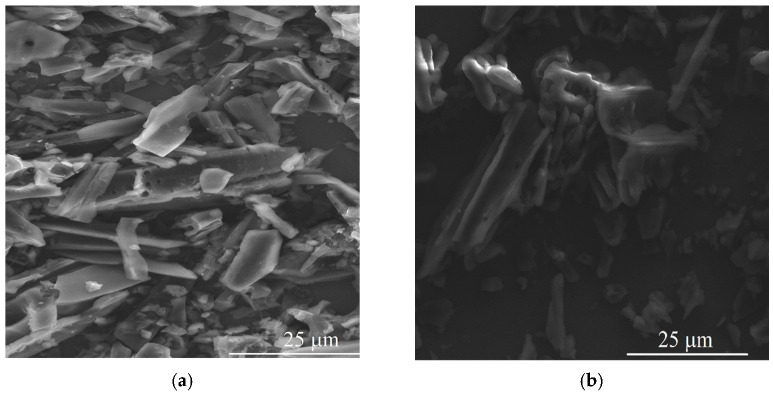
(**a**) SEM images of untreated RC; (**b**) and RC after acid functionalization.

The surface morphologies of RC and RCF were characterized by SEM, as displayed in Figure 2. Significant changes after acidic treatment were evaluated. As seen in Figure 2a, the typical structure of the untreated RC sample shows a heterogeneous form, with particles of random sizes and pores or honeycomb structures [27]. In Figure 2b, the RCF presents a noteworthy change in the structure of the material, with a decrease in pore volume and grooves on its surface. Similar observations have been reported in previous studies [28].

### 3.2. Electrochemical Characterization

**Figure 3:**
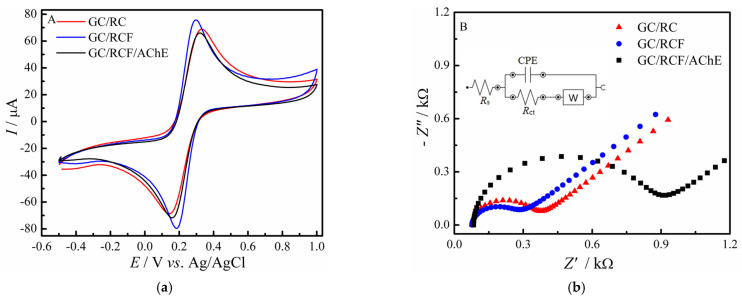
Electrochemical characterization of the electrodes in a 0.2 mol L^−1^ PBS (pH 7.4) solution having 0.1 mol L^−1^ of KCl and 5.0 mmol L^−1^ of the redox couple Fe[(CN)_6_]^3−/4−^ by (**a**) Cyclic Voltammetry; (**b**) Electrochemical Impedance Spectroscopy.

The electrochemical characterization of electrodes was carried out by experiments in in a 0.2 mol L^−1^ PBS (pH 7.4) solution having 0.1 mol L^−1^ of KCl and 5.0 mmol L^−1^ of the redox couple Fe[(CN)_6_]^3−/4−^. Figure 3a shows cyclic voltammograms profiles with well-defined oxidation and reduction peaks for the GC/RC (red line) and GC/RCF (blue line) modified electrodes. This behavior is due to the Fe^3+^/Fe^2+^ redox couple. The GC/RCF electrode showed a 1.4-fold increase in the peak current compared to the GC/RC electrode. This increase is due to the presence of defects introduced in its structure, as well the presence of functional groups. The GC/RCF/AChE biosensor presented a decrease in both anodic and cathodic currents due to the immobilization of AChE. 

The EIS experiments showed in Figure 3b are in accordance with the previous CV. The EIS experiment revealed that the resistance to charge transfer (*R*_ct_) for the electrodes was altered as the biosensor was fabricated. The GC/RC (▲) showed a *R*_ct_ of 311 Ω, after the functionalization step, the GC/RCF (●) electrode showed a decrease in the resistance charge, leading to a value of 257 Ω. Furthermore, the biosensor GC/RCF/AChE (■) showed a *R*_ct_ of 818 Ω, which is expected due to the presence of a poor conductor as an enzyme [7]. 

### 3.3. AChE Immobilization Process and Acetylcholine Iodide Oxidation

**Figure 4:**
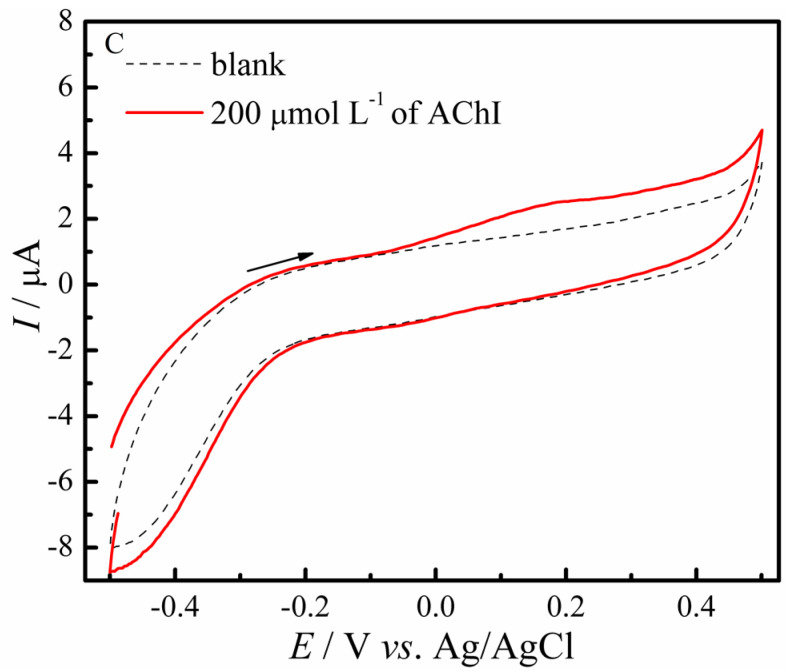
DPV response in the absence (dashed line) and presence (solid line) of 50.0 µmol L^−1^ of AChI using the GC/RC/AChE biosensor in 0.2 mol L^−1^ PBS solution (pH 7.5).

As reviewed in the literature, the AChE biosensor acts by exploring the inhibition of enzymatic activity by pesticides, predominantly by the carbamates and organophosphates classes [29]. The electrochemical characterization of the GC/RC/AChE biosensor in the presence of the substrate acetylthiocholine iodide (AChI) was carried out by CV experiments in 0.1 mol L^−1^ of PBS (pH 7.0). Figure 4 shows the voltammetric responses of the biosensor in the absence (dashed line) and in the presence (solid black line) of 200 μmol L^−1^ of AChI. In the absence of the substrate no electrochemical process was observed. On the other hand, in the presence of AChI (solid line), the proposed biosensor presented an oxidation peak at *E*_pa_ = +180 mV vs. Ag/AgCl/KCl (3.0 mol L^−1^). The investigation of this oxidation mechanism will be discussed next. 

**Figure 5:**
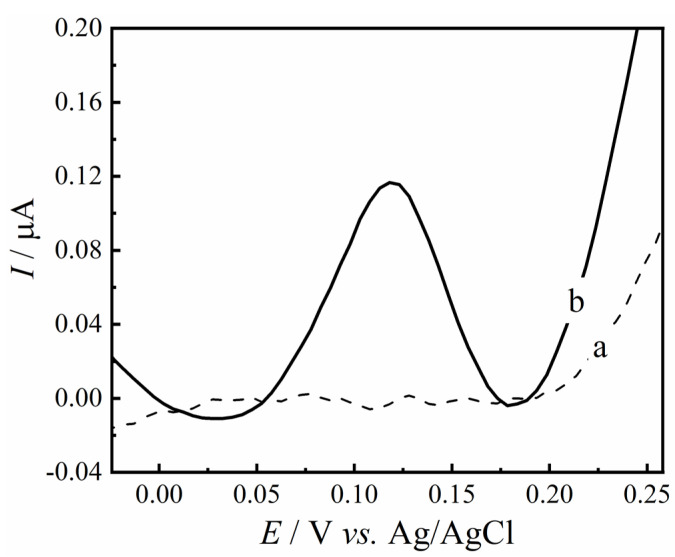
DPV response in the absence (a) and presence (b) of 50.0 µmol L^−1^ of AChI using the GC/RC/AChE biosensor in 0.2 mol L^−1^ PBS solution (pH 7.5).

As a complementary experiment, DPV measurements were conducted to quantify the oxidation process of the enzymatic reaction between the enzyme AChE and its substrate, AChI, responses of GC/RC/AChE biosensor shown in Figure 5. In the absence of AChI (curve a), no electrochemical process was observed. In contrast, the enzymatic immobilization process on the surface of the modified electrode is confirmed (curve b), even in the absence of an agglutinating agent such as glutaraldehyde or di-imide. As reported by Li et al. [30] RC, also known as biochar, presents several intrinsic characteristics that allow the correct immobilization of biomolecules and therefore its application in biosensing. Surface area, porosity, stability and the addition of surface functional groups through chemical treatment can contribute to the immobilization of AChE enzyme. The authors reported several works in literature with acid treatment for biochar to increase in of functional groups such as carboxylic and carbonyl group. In this context, the structure and the chemical groups present in the support material (RCF) may lead to immobilization of AChE enzyme through adsorption (Van der Walls force / Ionic interaction) and covalent bonding. The oxidation peak at approximately +100 mV is associated with the oxidation of thiocholine and the generation of its respective dimer, dithio-bis-choline (a hydrolysis byproduct of the neurotransmitter acetylcholine) [7,12,31].

(4) $$({\mathrm{CH}}_{3}{)}_{3}N^{+}{\mathrm{CH}}_{2}{\mathrm{CH}}_{2}{\mathrm{SCOCH}}_{3}\;+\;H_{2}O\;\overset{\mathrm{AChE}}{\to }\;({\mathrm{CH}}_{3}{)}_{3}N^{+}{\mathrm{CH}}_{2}{\mathrm{CH}}_{2}\mathrm{SH}\;+\;{\mathrm{CH}}_{3}\mathrm{COOH}$$

2(CH_3_)_3_N^+^CH_2_CH_2_SH → (CH_3_)_3_N^+^CH_2_CH_2_S-SCH_2_CH_2_N^+^(CH_3_)_3_ + 2e (5)

### 3.4. Optimization of GC/RCF/AChE Biosensor Response

#### 3.4.1. Effect of the Acid Functionalization Process

**Figure 6:**
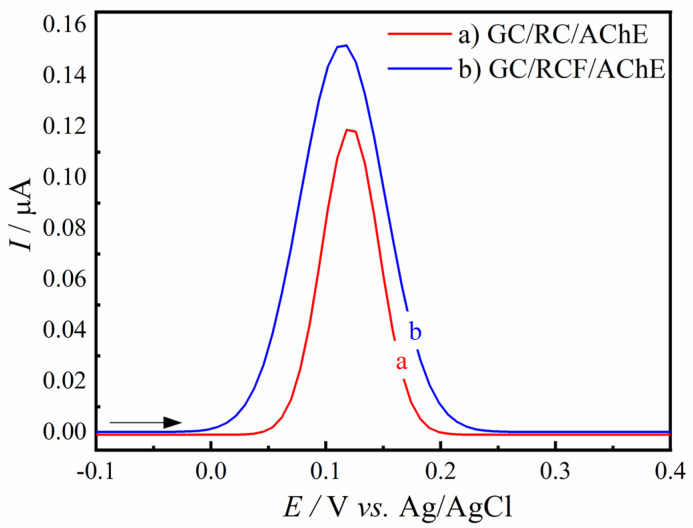
DPV responses of GC/RC/AChE (a) and GC/RCF/AChE (b) biosensors in the presence of 50.0 µmol L^−1^ of AChI substrate in a 0.2 mol L^−1^ PBS solution pH 7.5.

The effect of the acid functionalization process on the capability of renewable carbon to detect the oxidation peak of thiocholine was carried out. The response of the oxidation peak of thiocholine of the GC/RC/AChE and GC/RCF/AChE biosensors were compared, evidencing a 28% increase in the anodic peak current of the dimer response of the GC/RCF/AChE biosensor, as shown in Figure 6. Both materials were evaluated using 100 μg mL^−1^ of the AChE enzyme.

The acid functionalization/treatment of RC has been extensively applied in order to increase the presence of oxygenated organic groups as well as the oxygen to carbon ratio [25,32], and in some cases reduced porosity [33]. Such chemical modifications of this carbon-based material may present the RCF as a suitable platform for enzyme immobilization owing to adsorption (Van der Waals force or ionic interaction) and covalent bonding [32]. In the past decade, renewable carbon has been applied to successfully enzyme immobilization for a variety of applications, such as hydrolysis [34], enzymatic degradation [35] and molecule removal in wastewater treatment [36]. In addition, Martins et al. [37], developed a biochar-based electrochemical immunosensor for the selective detection of Hantavirus Araucaria nucleoprotein, known as a common virus of wild rodents that can cause hemorrhagic fever in humans after contamination. Activated biochar has also been reported in electroanalytical sensors towards bisphenol A [38,39] and ammonia detection [40].

#### 3.4.2. Optimization of RCF Concentration and pH of Supporting Electrolyte

**Figure 7:**
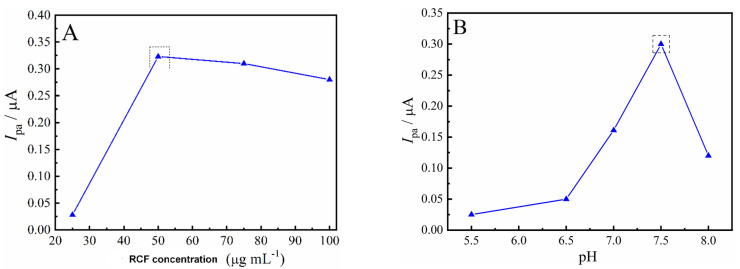
(**A**) Effect of RCF (µg mL^−1^) concentration and (**B**) effect of pH on the anodic peak current of thiocholine oxidation for the GC/RCF/AChE biosensor in a 0.2 mol L^−1^ PBS solution containing 50.0 µmol L^−1^ of AChI.

- For optimization purposes, the concentration of RCF during biosensor construction was evaluated between 25 to 100-μg mL^−1^, in PBS solution with 50 μmol L^−1^ of AChI, as shown in Figure 7A. The anodic peak current reaches its maximum value at the concentration of 50 μg mL^−1^, which was used in all the following experiments. In addition, the concentration of the AChE enzyme used was 40-µg mL^−1^, this value was optimized in a previous work of our research group [7].
- The effect of the supporting electrolyte’s pH on the anodic peak current was also studied by varying the pH from 5.5 to 8.0. Figure 7B shows that the oxidation of thiocholine reaches its maximum value at pH 7.5, which was previously reported in the literature as an optimum condition for the proper functioning of the AChE enzyme with no denaturation and consequently loss of its catalytic activity [41]. Therefore, pH 7.5 was chosen in all the subsequent experiments.

### 3.5. Analytical Curve of Carbaryl Pesticide

**Figure 8:**
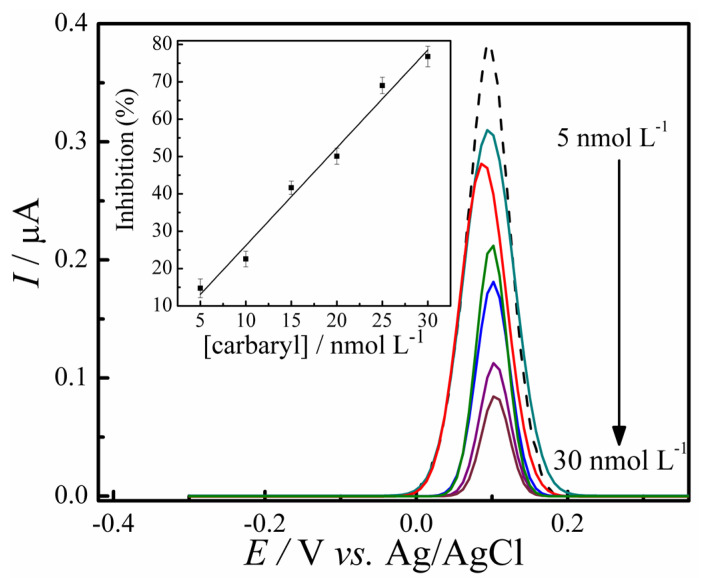
Baseline corrected DPV voltammograms for GC/RCF/AChE biosensor electrode in the absence (dashed line) and in the presence of 5.0–30.0 nmol L^−1^ of carbaryl. Inset: linear dependence of the enzyme inhibition with carbaryl concentrations.

The efficacy of carbaryl detection of GC/RCF/AChE biosensor was evaluated by the DPV voltammograms. The analytical curve was based on the difference of the oxidation current of thiocholine before and after adding different carbaryl concentrations, as described in the experimental section. The biosensor showed a linear response from 5.0 to 30.0 nmol L^−1^ as shown in Figure 8. Following the equation, I (%) = 1.5 × 10^−4^ + 2.62 × [carbaryl], with a correlation coefficient of R^2^ = 0.982 (*n* = 6). Following IUPAC recommendations, the limit of detection (LOD) of 4.5 nmol L^−1^ (0.91 ng mL^−1^) and a LOQ of 15.0 nmol L^−1^ (3.02 ng/mL). The developed method showed a sensitive response for carbaryl. The Brazilian Health Regulatory Agency (ANVISA) states that the maximum residue level (MRL) for apple crops is 2.0 mg/kg, and the Acceptable Daily Intake (ADI) is 0.003 mg per kg of body weight [6]. Considering the high toxicity and teratogen aspects of carbaryl, the development of biosensors able to detect this carbamate pesticide at low concentrations is fundamental.

Table 1 displays the comparison in analytical performance of GC/RCF/AChE biosensor with other recently reported electrochemical biosensors of carbaryl. Du et al. [42] synthesized a film of chitosan electrochemically deposited on the surface of the gold electrode combined with the AChE enzyme. The biosensor based on an electropolymerization polyaniline film for immobilization of the AChE enzyme in carbon nanotubes was developed by Cesarino et al. [43]. Loguercio et al. [44] optimized the surface conditions for the immobilization of the AChE enzyme using indigo carmine and dodecyl sulfate doped polypyrrole—gold nanoparticle nanocomposite films. In addition, the AChE-e-pGON/GCE and GC/rGO/AChE biosensors, developed, respectively, by Li et al. [45] and Silva et al. [7], which stood out among the biosensors with lower LODs for detecting the carbaryl pesticide.

Although other biosensors might present lower LODs, materials such as RCF validate the use of renewable carbon sources in the development of electrochemical biosensors with similar results for the detection of carbaryl pesticides and leading to low-cost analytical devices for agri-food safety [46]. 

The GC/RCF/AChE biosensor presented a greater sensitivity for the evaluation of the inhibition of the thiocholine (or inhibitory effect) signal caused by the addition of carbaryl, thus obtaining a linear dependence at a lower concentration range. As emphasized in the previous experiments, the acidic functionalization of carbonaceous materials, such as renewable carbon, tends to form functional groups on the material (mainly carboxylic, nitro, and hydroxylic groups), not only promoting a catalytic effect on dimer oxidation but also increasing the interaction properties between the renewable carbon and biomolecules such as AChE enzyme [47]. Enzymes tend to be immobilized on functionalized electrode surfaces, mainly through interactions such as adsorption, encapsulation, covalent bonding, and covalent cross-linking [8]. The renewable carbon used in this study has strong adsorption characteristics for organic molecules [48], with the presence of functional groups on its surface [49]. The synergistic effect of these two characteristics acted constructively to improve the thiocholine oxidation process, leading to greater sensitivity at low concentrations.

### 3.6. Influence of Interferent in GC/RCF/AChE Biosensor Performance

In order to evaluate the influence of possible interfering agents and investigate the selectivity of the proposed biosensor, DPV experiments were performed after the incubation of the biosensor in a PBS solution containing a previously defined aminomethylphosphonic acid (AMPA) concentration for five minutes. AMPA is the main metabolite of the herbicide glyphosate, with low inhibition of the AChE enzyme. As expected, the biosensor presented a good selectivity to the carbaryl pesticide, as the carbamate pesticide inhibited almost exclusively the oxidation process of dithio-bis-choline. On the other hand, when analyzing various concentrations of AMPA, the biosensor showed no significant change in the thiocholine oxidation process (figure not shown).

### 3.7. Lifetime, Repeatability and Reproducibility

The evaluation of the lifetime of the proposed biosensor was conducted by DPV experiments in the presence of 50.0 µmol L^−1^ of AChI (figure not shown). The GC/RCF/AChE biosensor showed similar responses to thiocholine oxidation in the period of 2, 3, 4, and 6 days. During the measurements, the biosensor was stored in 0.2 mol L^−1^ PBS solution (pH 7.5) at 4 °C. After 6 days in storage, a significant change in the electrochemical response of the thiocholine oxidation was observed. Although the active period of a renewable carbon-based biosensor seems to be shorter than the graphene-based one, renewable carbon is considered a low-cost and renewable material with a simpler synthesis [7]. 

The 9.3% repeatability for the proposed biosensor was obtained by measuring the current response of five DPV voltammograms after a five minute incubation in a solution with 50 µmol L^−1^ of AChI. The reproducibility of 9.7% was determined by the standard deviation of 5 sequential voltammograms from three experiments [50]. The repeatability and reproducibility of the GC/RCF/AChE biosensor showed higher values when compared to other electrochemical biosensors [12]. This may be associated with the fact that the proposed biosensor does not use crosslinking (glutaraldehyde) or bioconjugation agents, nor semiconductor polymers (polyaniline or polypyrrole) which increase the stability of the AChE enzyme, but on the other hand, would add complexity in the manufacture of the biosensor.

### 3.8. Analysis of Carbaryl in Apple Samples

**Figure 9:**
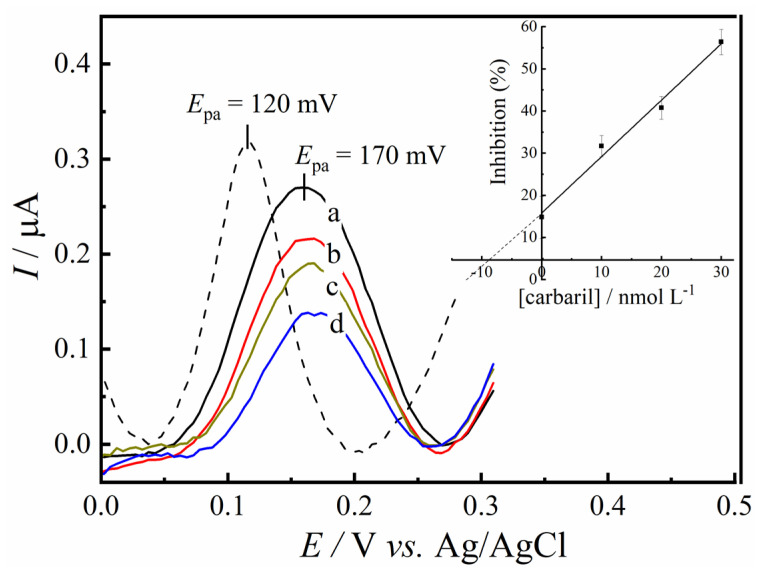
Proposed standard method addition for detecting carbaryl in apple samples. DPV responses in the absence (dashed line), apple sample fortified with 10.0 nmol L^−1^ of carbaryl (a), and spiked carbaryl concentrations of 10.0, 20.0, and 30.0 nmol L^−1^ (b–d).

This study evaluated the applicability of the developed biosensor in a real sample matrix, as described in Section 3.6. Even in a complex matrix, the GC/RCF/AChE biosensor showed a good response and linearity for carbaryl determination. However, it is worth noting that the anodic peak current of the thiocholine response shifted. This can be correlated to the matrix effect, observed after the addition of the sample fortified matrix with 10.0 nmol L^−1^ of carbaryl. The thiocholine oxidation shifted from 120 to ~170 mV, as shown in Figure 9. Similar behavior was also observed in the detection of carbaryl in tomato samples by Silva et al. (2018) [7]. 

For the construction of the response curve by the standard addition method, three successive additions of carbaryl were carried out in the 10.0, 20.0, and 30.0 nmol L^−1^ systems for the construction of the response curve by the standard addition method. Still, no significant change was observed in the determination of the pesticide concentration (inset Figure 9). 

The determination of carbaryl in apple samples was performed in triplicate, obtaining a mean value of 10.90 ± 0.90 nmol L^−1^. Recovery of added concentration ranged from 102.5 to 118.6%. The presence of the positive deviations obtained in the recovery of the added concentration may be associated with the presence of some constituent molecules of the apple, such as sugars, dietary fibers, and minerals (sodium and iron in particular).

## 4. Conclusions

The proposed GC/RCF/AChE electrochemical biosensor was successfully applied for the determination of Carbaryl pesticide in apple samples at lower levels stipulated by ANVISA. Although the biosensor has a relatively short lifespan, its low-cost character and simplicity of production bring great appeal to its users. The attribution of added value to a renewable material (produced from biomass residues) is a promise for future biosensor applications and is consistent with the aims of sustainable development.

For future research, it may be necessary to investigate different ways of enzyme immobilization techniques that can offer a greater electrode-enzyme interaction in order to preserve enzyme activity. In addition, other electrochemical techniques such as Square-wave Voltammetry or Chronoamperometry could be used in the investigation of such biosensors.

## Figures and Tables

**Table 1:** Comparison of analytical characteristics with other biosensors reported for carbaryl detection.

Method	Biosensor	Detection Range (nmol L^−1^)	LOD (nmol L^−1^)	Ref.
CV	AChE-CHIT/Au ^1^	25–500	14.9	[42]
DPV	GC/rGO/AchE	10–50	1.9	[7]
DPV	AChE-e-pGON ^2^/GCE	1.5–30.3	0.74	[45]
SWV ^3^	GC/MWCNT ^4^/PANI ^5^/AChE	9.9–49.6	4.6	[43]
Chronoamperometry	PPy-IC-DS1-AuNP-AChE	0.25–1.24	0.16	[44]
DPV	GC/RCF/AChE	5–30	4.5	This work

^1^ CHIT/Au: chitosan-modified gold electrode; ^2^ e-pGON: electrochemically induced porous graphene oxide network; ^3^ SWV: Square-Wave Voltammetry; ^4^ MWCNT: multiwalled carbon nanotubes; ^5^ PANI: polyaniline.

## Data Availability

The data is available under the request to the correspondence.
